# Screening for Familial Colorectal Cancer Risk amongst Colonoscopy Patients New to an Open-Access Endoscopy Center

**DOI:** 10.5402/2012/152980

**Published:** 2012-03-22

**Authors:** Sumana Moole, Thomas J. McGarrity, Maria J. Baker

**Affiliations:** ^1^Division of Gastroenterology & Hepatology, Department of Medicine, Penn State Hershey College of Medicine, Hershey, PA 17033-0850, USA; ^2^Division of Hematology/Oncology, Department of Medicine, Penn State Hershey Cancer Institute, Penn State Hershey College of Medicine, P.O. Box 850, Hershey, PA 17033-0850, USA

## Abstract

*Purpose*. We evaluated a questionnaire to aid in the recognition of CRC risk, as well as patient interest in their risk status within an open-access endoscopy center. *Methods*. A questionnaire was administered to new patients presenting for colonoscopy from May 2007 to February 2008. 287 patients were enrolled. Family history was evaluated using Amsterdam 1, II, and Revised Bethesda criteria. Recognition of risk and referral for counseling was assessed. Patients' interest to be contacted by a genetic counselor was also assessed. *Results*. 13.2 % (38/287) of patients met Revised Bethesda criteria. Of these, 18 (47.4 %) were previously told about their increased risk for CRC. Only 1 patient who met Revised Bethesda criteria (2.6 %) was previously referred for genetics, whereas none of the 3 patients who met Amsterdam I or II criteria were referred. 23.7 % of high-risk patients did not want to be contacted if found to be at increased risk for cancer. *Conclusion*. In our open-access endoscopy system, a significant number of high-risk patients remain unidentified and underreferred for genetic counseling due to numerous barriers. Our findings lend support to taking a public health approach to identifying those at risk for Lynch syndrome by implementing universal screening of all CRC specimens.

## 1. Introduction

Colorectal cancer (CRC) is the second most common cause of cancer-related death worldwide and represents a major health issue. The cumulative risk of getting and dying from CRC in the US is 6% and 2.5%, respectively. About 20–30% of colorectal cancer has a familial component, and 3–5% is due to an inherited syndrome [1].

Studies indicate the lifetime risk of developing CRC in an individual with a first-degree relative with CRC is increased by 1.6-fold to 8.0-fold [2, 3]. A meta-analysis by Johns and Houlston of 27 studies assessing familial CRC noted a relative risk of 2.25 in an individual with a first-degree relative with CRC. The relative risk doubled to 4.25 with more than one affected relative. Age at diagnosis was a strong risk factor, as well as having a young relative with a colorectal adenoma. This study provides strong evidence that both age at diagnosis and number of affected relatives are strong risk factors for familial CRC [2]. 

Survival in CRC is primarily related to the disease stage at the time of diagnosis. Only a minority (35%) of cases, though, are diagnosed with localized disease, and 20–25% are diagnosed with metastatic disease [4]. Overall five-year survival is 62% to 94% in individuals with localized disease. Screening or secondary prevention is defined as the presumptive identification of unrecognized disease by the application of tests. The biological basis for CRC screening is that the great majority of CRC arises from preexisting adenomatous polyps. Given the slow progression of colon polyps to cancer, screening is cost-effective and efficient in detecting early colorectal cancer. Of the various screening methods available, colonoscopy is the most sensitive with 95% sensitivity for the detection of colorectal cancer.

 Detection and removal of adenomatous polyps via screening has in part decreased the incidence of CRC from 38 cases per 100,000 in 1985 to 31 cases per 100,000 in year 2000 [4]. Similarly, CRC-related death rate per 100,000 has declined from 30.77 in 1990 to 21.57 in 2004. This is supported by the National Polyp Study which has shown a 76–90% decrease in CRC incidence after colonoscopic polypectomy compared with age-matched controls [5]. The long-term risk of CRC was reduced by endoscopic screening and surveillance [6–8].

 For the subset of CRC that is attributable to hereditary syndromes such as hereditary nonpolyposis colorectal cancer (HNPCC), or Lynch syndrome, and familial adenomatous polyposis (FAP), screening and surveillance recommendations differ significantly from those of the average risk population. It is crucial that physicians are aware of the guidelines and identify individuals and families at risk for hereditary CRC. Unfortunately, inadequate recommendations from physicians are recognized as a major barrier to CRC screening [9].

Open-access endoscopy (OAE) is a system that allows primary care physicians to directly access endoscopic procedures without a prior office visit. OAE promotes adherence to screening colonoscopy by increasing patients' convenience and decreasing patients' costs. Properly constructed, OAE provides efficient, appropriate endoscopy in a confidential and safe manner [10, 11]. A majority of gastroenterology practices offer OAE [12]. Few studies have looked at the appropriateness of referrals or recognition of high-risk syndromes in the referral population at such centers. The goal of this study is to evaluate the recognition of high-risk syndromes and subsequent referral for genetic counseling of individuals presenting to an open-access endoscopy center.

## 2. Methods

New patients between May 2007 and February 2008 who presented for colonoscopy at one of the two open-access endoscopy centers affiliated with the Penn State Milton S. Hershey Medical Center were presented with a voluntary family history questionnaire. See Table 1.

Patients were referred directly from community physicians, as well as from physicians within the university. The study was approved by the Human Subjects Protection Office and informed consent obtained from all patients.

Each consenting individual completed a 14-point questionnaire obtaining details regarding personal and family history of colorectal cancer/polyps, as well as other cancers. It also inquired about their willingness to be contacted by a genetic counselor if they were determined to be at high risk based on their family history. These forms were then analyzed by a genetic professional with the Penn State Hershey Cancer Genetics Program to determine if they met criteria to be evaluated for Lynch syndrome based on Amsterdam I, II, and Revised Bethesda criteria and their willingness to be contacted for consideration of genetic counseling and testing. Statistical analysis included calculation of simple percentages of the total.

## 3. Results

During the study period, a total of 1912 new patients presented to the Hershey Endoscopy Center (HEC) for colonoscopy. An additional 806 new patients presented to the University Physician Center (UPC) Endoscopy Suite during the same time period. 328 patients combined from both study sites participated in the study. 41 questionnaires, however, were eliminated due to the presence of more than one incomplete answer. A total of 287 questionnaires were used for the final data analysis. Of the 287 questionnaires successfully completed, 160 were filled out by patients seen at HEC for a participation rate of 160/1912 or 8.4%. 104 questionnaires were successfully completed by patients seen at the UPC Endoscopy Suite for a participation rate of 104/806 or 12.9%. For 23 of the surveys, it was not certain at which site the patient presented for colonoscopy. See Table 2 for demographics of patients included in the final analysis. Only 3/287 (1.0%) patients within the study population met criteria for Lynch syndrome, based on Amsterdam I or II criteria, whereas 38/287 (13.2%) had personal and/or family history that met Revised Bethesda criteria such that MSI testing on a CRC specimen would be indicated. See Table 3 for tabulated results.

Of the 38 individuals who met Amsterdam I, Amsterdam II, or the Revised Bethesda criteria, 26 (68.4%) expressed interest in being contacted if determined to be at increased risk, 9 (23.7%) did not want to be contacted, and 3 (7.9%) did not answer the question. Of the 26 patients who expressed interest in being contacted, 24 (92.3%) were notified regarding their increased risk by a telephone call from a genetics professional. The remaining 2 patients who wanted to be contacted did not return our calls. Of all patients who successfully completed the survey, regardless of their level of risk, 145/287 (50.5%) were willing to be contacted by a genetic counselor if they were determined to be at increased risk for a hereditary cancer syndrome, 30.0% or 86/287 did not want to be contacted, and 56 (19.5%) did not answer the specific question.

Interestingly, 25/287 (8.7%) patients who completed the survey as part of their visit to one of the open-access endoscopy centers met criteria for referral to a genetic counselor to discuss genetic testing for hereditary breast and ovarian cancer (HBOC) syndrome which is associated with mutations in the BRCA1 and BRCA2 genes. Of these 25 patients, 15 (60.0%) had given permission to be contacted if they were determined to be at increased risk for a genetic predisposition to cancer but 3 did not return our calls. An additional 2 patients (0.7%) had a prior diagnosis of familial adenomatous polyposis (FAP).

The 2008 joint guidelines of the American Cancer Society, the US Multi-Society Task Force on Colorectal Cancer, and the American College of Radiology recommend screening of patients beginning at age 40 with a family history of one first degree relative with adenomatous polyps or cancer at any age [13]. Of the first-time colonoscopy takers in our study population which represented 136/287 or 47.4% of those completing a family history questionnaire, 46/136 (33.8%) had a first-degree relative with polyps and 16/136 (11.8%) had a first-degree relative with CRC which warranted baseline screening at 40. 4.4% (6/136) had a history of both CRC and polyps in a first degree relative(s). Of the 56 patients who had either a first degree relative with CRC and/or polyps where earlier screening was warranted, 44 (78.6%) were above the age of 40 at the time of their first colonoscopy.

## 4. Discussion

Colorectal cancer typically occurs in individuals older than 50 owing to age-related associated risk and environmental and/or lifestyle-related exposures [14]. Studies have shown that 20–30% of CRC has a potentially identifiable genetic cause [2]. Specific genes have been identified as the causative factors for high-risk syndromic CRC. Amongst the high-risk syndromes, the most common are Lynch syndrome and FAP, which are caused by germ line mutations in the associated genes and account for 3–5% of all CRC [14–16]. Genetic testing can reveal mutations in high-risk individuals and their family members and help guide further screening recommendations. To adequately detect the subset of patients with Lynch syndrome, genetic testing should be considered in approximately 10–15% of all patients with CRC [17, 18].

Additionally, sporadic colon cancer often displays clustering in families as well, though not necessarily meeting criteria for well-defined genetic syndromes. These sporadic cases are thought to arise due to low-penetrance genes with a number of candidate genes and polymorphisms already identified [14, 19–21]. Current National Comprehensive Cancer Network (NCCN) guidelines to screen such individuals with familial clustering who do not meet specific criteria include (1) to begin screening at age of 40 years or 10 years prior to diagnosis and subsequently every 3–5 years if one first-degree relative <50 yrs is affected. (2) Screening at 40 and repeat every 3–5 years if 2 or more first-degree relatives at any age or one younger than 50 are affected [13, 22]. The age limit of 50 has further been liberalized to 60 to be considered in the increased risk category for CRC by the joint guidelines. They recommend beginning screening at 40 and then every 5 years if 1 first-degree relative younger than 60 or two relatives over the age of 60 is affected with CRC.

Our study showed that a significant number of newcomers to colonoscopy (41.2%) met criteria to warrant earlier screening starting at 40 due to the presence of at least one first-degree relative with CRC or polyps according to the joint guidelines. By this measure, 44/56 (78.6%) were screened late. We acknowledge, though, that these guidelines were released in early 2008.

A study of 535 CRC patients indicated that selectively testing CRC patients with high-risk features, including age less than 50, presence of multiple primary cancers, affected first-degree relatives with CRC or endometrial cancer, significantly improved efficiency. These form part of the Bethesda guidelines put forth by the National Cancer Institute Workshop on HNPCC in 1997 [23, 24].

All of the above criteria, though, rely on a thorough personal and family history to aid in the recognition of high risk syndromes and the appropriate referral of patients for genetic counseling. Our study shows that only 18 of the 38 patients whose family history met the Revised Bethesda criteria (47.4%) were recognized appropriately as high risk by the referring physicians, indicating either inadequate history uptake or lack of sufficient knowledge regarding the clinical criteria.

A newer entity known as MYH-associated polyposis (MAP), which is due to germline mutations in the MYH gene, usually presents in the 4th and 5th decade and follows an autosomal recessive mode of inheritance. Affected individuals tend to have fewer polyps (less than 100) than the classic form of FAP and are at risk for developing CRC, as well as duodenal cancer, similar to those with FAP. Our family history questionnaire did not ask the cumulative number of polyps identified in patients and their relatives due to this information typically not being known. As a result, it was difficult to distinguish concern for Lynch syndrome versus one of the polyposis syndromes, such as FAP or MAP, if there was only a history of CRC/polyps in the absence of Lynch-associated cancers.

Although not one of the goals of our study, the questionnaire also helped identify families that may be at increased risk to harbor a BRCA mutation. The criteria that were used to determine if a family was appropriate to consider BRCA counseling and testing are as follows: (1) 2 or more women on the same side of the family with breast cancer <50, (2) any woman in the family with both breast and ovarian cancer, (3) 2 primary breast cancers in the same woman, (4) any woman with Ashkenazi Jewish ancestry and a history of breast or ovarian cancer, (5) any male with breast cancer, (6) a woman with breast cancer in her 20s or 30s regardless of any additional family history, and (7) at least 3 women on the same side of the family with breast cancer regardless of age at diagnosis. A total of 8.7% (25/287) of the study population met criteria which raised concern for a hereditary predisposition to breast and ovarian cancer but only 1/25 or 4% had previously been referred for genetic counseling. Similar to those patients at increased risk for Lynch syndrome, as identified by meeting the Revised Bethesda criteria, a substantial portion of patients were unaware of their potential increased risk for hereditary breast and ovarian cancer syndrome at the time of presentation for colonoscopy.

Increasingly, open-access endoscopy (OAE) is viewed as a way to increase access, improve convenience, and decrease costs of CRC screening. The challenge to OAE is to provide safe and appropriate screening. A checklist format encompassing established guidelines for average and high-risk individuals is critical for OAE quality control.

Implementing the family history form uncovered several interesting points, in addition to the limited recognition of familial cancer within the medical community. Further, it brought to light the underutilization of genetic counseling, as well as individual attitudes towards such referrals. Only 2.6% of the study population that met criteria concerning Lynch syndrome were referred for genetic counseling, and, interestingly, 29.8% of the high-risk patients elected not to be contacted if determined to be at increased risk.

Approximately, 10–15% of all patients with CRC should be considered for genetic testing to identify most cases of Lynch syndrome. A study by Grover et al. noted that 19% (75/387) of patients enrolled in the study met criteria for MSI testing but only 17% (17/75) of the individuals meeting criteria were tested [18]. Our results likewise show that a significantly small number of patients undergo genetic counseling/testing compared to the actual number of patients who met referral criteria. Possible barriers include identification and referral of appropriate patients by physicians, physician knowledge regarding familial syndromes, and the various societal guidelines, as well as patient access to genetic services. In addition, patients may have limited knowledge regarding the potential benefits of one's genetic information, and they may fear the potential risk of genetic discrimination and have concerns regarding psychological and emotional adjustment to one's genetic test results [25–27].

One of the major limitations of the study was that a significant number of patients elected not to complete the questionnaire and, of those that did, a fair number failed to answer the questions completely. Some of the possible reasons could be the length of the questionnaire, as well as the use of open-ended questions, both of which increase the amount of time required to complete the survey. Simplifying the family history form should help to obtain a higher yield as demonstrated by Kastrinos et al. in their open access endoscopy study [28]. Using recursive partitioning analysis, they developed a simple CRC risk assessment tool consisting of 3 questions that were most informative for identifying high-risk patients. The 3 questions consisted of (i) “Do you have a first degree relative with CRC or Lynch syndrome-related cancer diagnosed before age 50?” (ii) “Have you had CRC or polyps diagnosed before age 50?” (iii) “Do you have ≥3 relatives with CRC?” When asked successively, these questions identified 77% of high-risk patients and 95% of known mutation carriers [28]. Similarily, Nathanson et al. developed a 3-question risk assessment tool which helped to better identify patients at increased risk for CRC as evidenced by 24% of average risk patients having adenomatous polyps in comparison to the 41% of high-risk patients [29]. The 3 questions consisted of (i) “Do you have a history of colonic polyps or CRC?” (ii) “Do you have a family history of CRC?” (iii) “Have you or has anyone in your family had cancer of the uterus, ovary, stomach, intestines, or kidneys?” Although our study demonstrated that use of a family history questionnaire can increase the identification of high-risk patients, we did not have approval from the Human Subjects Protection Office to access the results of the colonoscopy and any biopsies taken to determine whether they had a higher percentage of CR polyps/cancer detected than those at average risk. As shown by our study and others [28, 29], family history forms can significantly increase the detection of high-risk patients. Kastrinos et al. concluded that approximately 1 in 5 patients undergoing colonoscopy would benefit from further risk assessment. Additionally, depending on the questions utilized in the risk assessment tool, concern for other cancer predisposition syndromes, such as Hereditary Breast and Ovarian Cancer syndrome can be raised as well.

Finally, an ample number of studies have investigated physician knowledge of familial CRC syndromes, screening and practice patterns [30–33]. They unanimously note suboptimal knowledge of CRC screening guidelines and criteria for familial syndromes. Interestingly, even within the subspecialty of gastroenterology, though the majority of gastroenterologists do obtain a family history, only a fraction has typically recommended genetic counseling and appropriate screening. Physician knowledge can thus prove to be a significant barrier to maximizing the benefits of genetic counseling and testing. Moreover, the lack of ample time in the all-too-familiar busy clinic schedule may, in part, attribute to inadequacies in history taking. Therein lies one of the challenges of an open-access endoscopy center where patients present for predetermined procedures without prior examination by the physicians performing those procedures.

These study findings support the necessity of identifying alternative strategies to gathering adequate family history in a system pressed for time so as to identify high-risk families. Given the various barriers which persist, though, all of which prevent high-risk patients from accessing genetic services, including both counseling and testing, a public health approach to screening all CRC cases for Lynch syndrome has been advocated by EGAPP, the Evaluation of Genomic Applications in Practice and Prevention Working Group [34]. These two approaches, utilization of a simplified clinical question set and universal screening of all CRC cases for Lynch syndrome, should be viewed as complementary to one another, thus permitting the greatest detection of patients and families with both familial and hereditary colorectal cancer.

## Figures and Tables

**Table 1:** Hereditary Colorectal Cancer Syndrome Survey (IRB number 23613).

Does anyone in your family have colon POLYPS?		
Relationship to you	Mother or Father's side of family	Age at diagnosis
_________________________________________________________________________________________________________________________________________________________		
_________________________________________________________________________________________________________________________________________________________		
_________________________________________________________________________________________________________________________________________________________		
Does anyone in your family have COLON or RECTAL cancer?		
Relationship to you	Mother or Father's side of family	Age at diagnosis
_________________________________________________________________________________________________________________________________________________________		
_________________________________________________________________________________________________________________________________________________________		
_________________________________________________________________________________________________________________________________________________________		
Does anyone in your family have UTERINE cancer?		
Relationship to you	Mother or Father's side of family	Age at diagnosis
_________________________________________________________________________________________________________________________________________________________		
_________________________________________________________________________________________________________________________________________________________		
_________________________________________________________________________________________________________________________________________________________		
Does anyone in your family have STOMACH cancer?		
Relationship to you	Mother or Father's side of family	Age at diagnosis
_________________________________________________________________________________________________________________________________________________________		
_________________________________________________________________________________________________________________________________________________________		
_________________________________________________________________________________________________________________________________________________________		
Does anyone in your family have PANCREATIC cancer?		
Relationship to you	Mother or Father's side of family	Age at diagnosis
_________________________________________________________________________________________________________________________________________________________		
_________________________________________________________________________________________________________________________________________________________		
_________________________________________________________________________________________________________________________________________________________		
Does anyone in your family have OVARIAN cancer?		
Relationship to you	Mother or Father's side of family	Age at diagnosis
_________________________________________________________________________________________________________________________________________________________		
_________________________________________________________________________________________________________________________________________________________		
_________________________________________________________________________________________________________________________________________________________		
Does anyone in your family have BRAIN cancer?		
Relationship to you	Mother or Father's side of family	Age at diagnosis
_________________________________________________________________________________________________________________________________________________________		
_________________________________________________________________________________________________________________________________________________________		
_________________________________________________________________________________________________________________________________________________________		
Does anyone in your family have URETER/KIDNEY cancer?		
Relationship to you	Mother or Father's side of family	Age at diagnosis
_________________________________________________________________________________________________________________________________________________________		
_________________________________________________________________________________________________________________________________________________________		
_________________________________________________________________________________________________________________________________________________________		
Does anyone in your family have SMALL BOWEL cancer?		
Relationship to you	Mother or Father's side of family	Age at diagnosis
_________________________________________________________________________________________________________________________________________________________		
_________________________________________________________________________________________________________________________________________________________		
_________________________________________________________________________________________________________________________________________________________		
		

Does anyone in your family have any OTHER cancers not listed (ex. Hepatoblastoma, a childhood liver cancer; cancers of the bile duct or gallbladder, etc.)		
Relationship to you	Mother or Father's side of family	Age at diagnosis
_________________________________________________________________________________________________________________________________________________________		
_________________________________________________________________________________________________________________________________________________________		
_________________________________________________________________________________________________________________________________________________________		
Did one of the following providers refer you for your colonoscopy? If yes, circle the provider. If not, circle other.		
McGarrity, Mathew, Ouyang, Riley, Pooran, Rampertab, Smith, Bethards, Schreibman, Meitz, Mukherjee, Downey, Moyer, Biswas, Moole, Thompson, Chase		
Other		
What is your age?		
Have you ever had a colonoscopy before (*circle*) Yes No		
If yes, when		
What were the findings:		
Why were you referred for today's colonoscopy?		
Have you ever been told that you are at an increased risk for colon or rectal cancer? (*circle*) Yes No		
If yes, reason given to you		
Do you have a personal history of these cancers: (*circle*):		
Colon, rectum, uterine, stomach, pancreas, ovarian, brain, gallbladder, ureter/kidney, small bowel, bile duct, breast, thyroid or any other cancers?		
Age at diagnosis		
Does cancer run in your family? For each individual, list type of cancer, and estimated age when the cancer was found		
If person has multiple cancers, please list them all.		
	*Type of cancer*	*Age at diagnosis*
Mother		
Father		
Siblings		
Grandmother (father's side)		
Grandmother (mother's side)		
Grandfather (father's side)		
Grandfather (mother's side)		
Aunts (indicate mother or father's side)		
Uncles (indicate mother or father's side)		
Nieces or Nephews		
Cousins (indicate mother or father's side)		
Your children		
Have you been told by your doctor that you may be at increased risk of a hereditary cancer syndrome?		(*circle*) Yes No
If so, have you been referred to a genetics counselor by your doctor?		(*circle*) Yes No
If it is determined that your family may be at increased risk for a genetic predisposition to cancer		
do you want to be contacted by Dr. Maria Baker, a genetics counselor with the Penn State Cancer Genetics Program?		(*circle*) Yes No

All information will be kept confidential and not released without your signed permission.

**Table 2:** Patient Demographics (*N* = 287).

Variable	*N*	%	Mean
Gender			
Male	93	32.4	
Female	170	59.2	
Not specified	24	8.4	
Age in years			54.4
<40	36	12.5	
40–49	43	15.0	
50–59	108	37.6	
≥60	90	31.4	
Not specified	10	3.5	
Referral indication			
Family history	62	21.6	
Routine screening	56	19.5	
Personal history CRC/polyps	47	16.4	
Bleeding/anemia	27	9.4	
Abdominal pain/hemorrhoids/stool changes	20	7.0	
Inflammatory bowel disease	14	4.9	
Other	11	3.8	
Not specified	50	17.4	

**Table 3:** Identification of patients with hereditary/familial risk factors (*N* = 287, *n* = 136).

Risk criterion met	*N*	%	*n*	%	Late to 1st c-scope	%
Hereditary risk criterion met by patient						
Amsterdam I criteria	1	0.3				
Amsterdam II criteria	3	1.0				
Revised Bethesda guidelines	38	13.2				
Familial risk criterion met by patient						
Px hx of colorectal polyps	39	13.6				
Px hx of colorectal cancer	6	2.1				
Fx hx of colorectal polyps	100	34.8				
Fx hx of colorectal cancer	94	32.8				
Fx hx of CR polyps in 1st degree relative(s)	93	32.4				
Fx hx of CR cancer in 1st degree relative(s)	49	17.1				

Familial risk criterion met by newcomer to colonoscopy						
Fx hx of CR polyps in 1st degree relative(s)			46	33.8	38	82.6
Fx hx of CR cancer in 1st degree relative(s)			16	11.8	11	68.8
Fx hx of either CR polyps and/or CRC in 1st degree relative(s)			56	41.2	44	78.6

Px hx: personal history, Fx hx: family history.
